# Factors influencing delayed lactogenesis II among advanced-age women following cesarean section: a retrospective analysis and predictive model development

**DOI:** 10.3389/fmed.2026.1733012

**Published:** 2026-01-20

**Authors:** Lei Chen, Xia Yu, Xiangding Wang, Hong Hu, Lu Zhang

**Affiliations:** 1 Department of Obstetrics, Luzhou Maternal and Child Health Hospital, Luzhou, Sichuan, China; 2 Department of Child Health Care, Luzhou Maternal and Child Health Hospital, Luzhou, Sichuan, China

**Keywords:** advanced maternal age, breastfeeding, cesarean section, delayed lactogenesis II, lactation, predictive model

## Abstract

**Background:**

Delayed lactogenesis II (DLII), defined as the onset of copious milk secretion after 72 h postpartum, is a common complication that may impair early breastfeeding success. Advanced maternal age and cesarean delivery are both known risk factors, yet few studies have developed predictive models to identify high-risk women. This study aimed to construct and internally validate a clinical prediction model for DLII among advanced-age women undergoing cesarean section.

**Methods:**

This retrospective observational study analyzed the medical records of 325 women aged ≥35 years who underwent cesarean delivery at a tertiary maternal and child health hospital in Southwest China between January 2021 and January 2025. Maternal, obstetric, surgical, and neonatal variables were extracted from electronic medical records. Univariate and multivariate logistic regression analyses were performed to identify independent predictors of DLII, and a nomogram was subsequently developed. Model discrimination and calibration were assessed using the area under the receiver operating characteristic (ROC) curve (AUC), Hosmer–Lemeshow goodness-of-fit test, and calibration plots with 1,000 bootstrap resamples.

**Results:**

Of the 325 women included, 117 (36.0%) experienced DLII. Multivariate analysis identified maternal age (aOR = 1.15, 95% CI 1.01–1.32), pre-pregnancy body mass index (BMI; aOR = 1.10, 95% CI 1.00–1.22), primiparity (aOR = 1.74, 95% CI 1.02–2.96), and shorter gestational age (aOR = 0.71, 95% CI 0.55–0.91) as independent risk factors for DLII. In contrast, elective cesarean section and early postpartum practices (rooming-in within 24 h, breastfeeding initiation within 24 h, and early skin-to-skin contact) were associated with lower odds of delayed lactogenesis II after multivariable adjustment (all *p* < 0.05). The final model demonstrated excellent discrimination (AUC = 0.870, 95% CI 0.830–0.910) and good calibration (Hosmer–Lemeshow *χ*^2^ = 5.60, *p* = 0.692).

**Conclusion:**

This study established a reliable and clinically interpretable model for predicting delayed lactogenesis II among advanced-age women undergoing cesarean section. The model showed high accuracy and good internal validity, highlighting the relevance of maternal characteristics and early postpartum behaviors to lactation outcomes. The proposed nomogram may serve as a practical tool for early risk identification and targeted breastfeeding support in clinical settings.

## Introduction

In recent decades, global demographic shifts have led to a marked increase in the proportion of women choosing to bear children at an advanced maternal age, typically defined as 35 years or older (1). This trend is particularly pronounced in urbanized regions where sociocultural and economic factors have contributed to the postponement of childbearing (2). Concurrently, the prevalence of cesarean section deliveries remains high and, in some populations, continues to rise (3). These epidemiological changes have brought new challenges to perinatal care, especially regarding breastfeeding initiation and maintenance, which are critical for optimal neonatal growth, immune protection, and long-term health outcomes (4). Delayed onset of lactogenesis II (DLII), commonly manifested as the late establishment of copious milk secretion postpartum, represents a significant barrier to successful breastfeeding in this vulnerable group, with potential repercussions for both maternal and infant wellbeing (5).

Epidemiological studies have demonstrated that advanced maternal age and cesarean delivery are independently associated with an increased risk of DLII (6, 7). The incidence of DLII in the general obstetric population varies, but data suggest that rates are notably higher among older mothers and those undergoing surgical delivery (8). Current literature indicates that DLII can exacerbate challenges in early breastfeeding, heighten the risk of neonatal dehydration, hypoglycemia, and hospital readmission, and contribute to early cessation of exclusive breastfeeding (9). Additionally, DLII may intensify maternal anxiety and undermine confidence in breastfeeding, thereby perpetuating a cycle of suboptimal infant feeding practices (10). The compounded effects of advanced age and cesarean section underscore the need for focused investigation and targeted intervention strategies.

Despite widespread recognition of the health benefits conferred by early and sustained breastfeeding, clinical management of DLII remains suboptimal, particularly in high-risk groups such as older women undergoing cesarean section (11). Previous research has identified several risk factors for DLII, including elevated pre-pregnancy body mass index (BMI), primiparity, shortened gestational age at delivery, peripartum complications, and delayed mother-infant contact (12). However, most existing studies have focused on isolated risk factors or on the general obstetric population, thereby limiting the applicability of their findings to high-risk subgroups. Moreover, current clinical practice often relies on empirical judgment rather than systematic risk assessment, resulting in missed opportunities for early identification and intervention (13). However, few studies have simultaneously examined maternal characteristics, surgical factors, and early mother–infant interactions within a single, integrated model to assess the individualized risk of delayed lactogenesis II among advanced-age women undergoing cesarean delivery (8).

Given these gaps, there is an urgent need to develop and validate robust, evidence-based predictive tools tailored to the unique clinical profile of older women undergoing cesarean section. Contemporary approaches to risk prediction in obstetrics have increasingly emphasized the utility of multivariable models that synthesize diverse clinical parameters, thereby facilitating individualized risk stratification and targeted clinical decision-making (14). To date, however, few studies have systematically integrated maternal demographic, obstetric, perioperative, and early postnatal behavioral variables into a predictive model for DLII specifically within the context of advanced maternal age and cesarean delivery. Furthermore, little is known about the external validity and practical applicability of such models in different regional and institutional settings, particularly in rapidly developing healthcare systems.

Accordingly, this study aimed to identify the multifactorial determinants of delayed lactogenesis II among women of advanced maternal age undergoing cesarean section and to develop and internally validate a clinically applicable risk prediction model to support early risk stratification and targeted breastfeeding interventions.

## Methods

### Study design and setting

This retrospective cross-sectional study was conducted at the Department of Obstetrics of a tertiary maternal and child health hospital in Southwest China. The hospital serves as a regional referral center for obstetric and neonatal care. Medical records of advanced-age women (aged ≥35 years) who underwent cesarean section between January 2021 and January 2025 were reviewed. The study adhered to the principles of the Declaration of Helsinki and was approved by the institutional ethics committee of the participating hospital (Approval No. LZFY-KL-2025-32); the requirement for informed consent was waived due to the retrospective design.

### Participants

Eligible participants were women aged ≥35 years who delivered singleton, live-born infants via cesarean section during the study period. Exclusion criteria included: (1) preterm delivery (<37 gestational weeks), (2) multiple pregnancy, (3) fetal congenital anomalies, (4) severe obstetric complications such as eclampsia, placenta accreta, or postpartum hemorrhage >1,000 mL, (5) pre-existing endocrine disorders (e.g., thyroid or pituitary disease), (6) incomplete clinical or lactation records, and (7) no intention to breastfeed or documented contraindications to breastfeeding (when available in the medical records). A total of 325 women met the inclusion criteria and were included in the final analysis. Participants were stratified into two groups according to the timing of lactogenesis II onset: normal (<72 h postpartum) and delayed (>72 h postpartum). Lactogenesis II timing was assessed during routine postpartum hospitalization and documented by attending midwives/lactation consultants; maternal report was corroborated using standardized nursing and lactation records.

### Variables and outcome definitions

The primary outcome of this study was delayed lactogenesis II (DLII), defined as the onset of copious milk secretion occurring later than 72 h postpartum, as determined by maternal report and confirmed through nursing and lactation records. Women who never initiated breastfeeding during the postpartum hospitalization were excluded whenever such information was documented. Predictor variables were identified based on prior evidence and clinical relevance, encompassing maternal, intrapartum, surgical, and neonatal characteristics. Maternal factors included age, pre-pregnancy body mass index (BMI), parity, gestational age, pregnancy-induced hypertension (PIH), and gestational diabetes mellitus (GDM). Intrapartum and surgical factors comprised the type of cesarean section (elective or emergency), anesthesia type, duration of operation, and estimated blood loss. Elective cesarean section was defined as a planned procedure scheduled before the onset of labor and before rupture of membranes, performed for non-urgent maternal or fetal indications. Emergency cesarean section was defined as an unplanned intrapartum procedure performed after labor onset and/or rupture of membranes because of an urgent maternal or fetal condition requiring expedited delivery. Commonly reported indications for elective cesarean section include a history of prior cesarean delivery, malpresentation (e.g., breech), placenta previa/low-lying placenta, suspected macrosomia, and cesarean delivery on maternal request; common indications for emergency cesarean section include nonreassuring fetal heart rate patterns, arrest disorders (failure to progress), failed induction of labor, placental abruption, and umbilical cord prolapse (15). Postpartum and neonatal factors included the 1-min Apgar score, rooming-in within 24 h, initiation of breastfeeding within 24 h, and early skin-to-skin contact. All variables were retrospectively obtained from the hospital’s electronic medical record system and verified by two independent investigators who were blinded to the study hypothesis to ensure data accuracy and consistency.

### Data sources and measurements

Clinical data were extracted from the hospital’s obstetric electronic medical record system. Maternal characteristics (age, parity, pre-pregnancy BMI) were obtained from prenatal records. Perioperative data (gestational age, operation time, blood loss) were derived from operative and anesthesia reports. Neonatal outcomes (Apgar scores, birth weight) and feeding-related behaviors (rooming-in, skin-to-skin contact, breastfeeding initiation) were documented by ward nurses and lactation consultants according to standardized protocols. The 5-min Apgar score was not consistently available in a structured format in the electronic record system during the study period and was therefore not included in the present analyses. To ensure data quality, two investigators independently extracted data using a standardized abstraction form. A random 10% sample of included records was then re-abstracted by a second investigator who was blinded to the outcome status. The re-abstracted variables included the primary outcome and all candidate predictors. Discrepancies were resolved by reviewing the original electronic medical records and reaching consensus; if disagreement persisted, a third senior investigator adjudicated.

### Bias control

To minimize selection bias, all eligible advanced-age cesarean deliveries within the study period were consecutively included. Measurement bias was reduced through the use of uniform documentation standards across the obstetric ward. Potential confounders identified from prior literature (e.g., parity, gestational age, and hypertensive disorders) were adjusted in the multivariate regression model. Data extraction and quality control were performed independently by two investigators to ensure consistency.

### Study size

A sample size justification was performed using G*Power (version 3.1.9.7; Heinrich Heine University Düsseldorf, Düsseldorf, Germany) to ensure adequate statistical power for the main analyses, based on the anticipated effect size and observed outcome incidence. Given the observed incidence of delayed lactogenesis II (36.0%) among the study population, a sample of 325 participants provided a statistical power greater than 0.90 to detect an odds ratio of 1.5 for the association between key predictors (such as primiparity or elevated pre-pregnancy BMI) and DLII at a two-sided *α* level of 0.05. This sample also satisfied the recommended criterion of at least 10 outcome events per variable (16), confirming that the dataset was sufficient for reliable multivariable logistic regression modeling.

### Quantitative variables

Continuous variables were assessed for normality using the Shapiro–Wilk test. Normally distributed data were presented as mean ± standard deviation (SD), whereas skewed data were expressed as median (interquartile range, IQR). For regression modeling, continuous predictors (e.g., maternal age, BMI, operation time, blood loss, gestational age) were treated as linear variables after verifying no significant nonlinearity.

### Statistical analysis

All statistical analyses were conducted using R software (version 4.3.3, R Foundation for Statistical Computing, Vienna, Austria). Continuous variables were assessed for normality using the Shapiro–Wilk test and presented as mean ± standard deviation (SD) or median with interquartile range (IQR), as appropriate. Group comparisons between women with and without delayed lactogenesis II (DLII) were performed using the independent-samples *t* test or Mann–Whitney U test for continuous variables and the *χ*^2^ test for categorical variables. Variables with *p* < 0.10 in univariate analysis were entered into a multivariate logistic regression model using a backward stepwise selection method to identify independent predictors of DLII. Early postpartum care variables were included to account for differences in breastfeeding-supportive practices during hospitalization; therefore, estimates from the adjusted model reflect associations conditional on these practices. The strength of association was expressed as odds ratios (ORs) and adjusted odds ratios (aORs) with corresponding 95% confidence intervals (CIs). A predictive nomogram was then constructed based on the final multivariable model to facilitate individualized risk estimation. Model performance was evaluated in terms of discrimination and calibration. Discrimination was quantified by the area under the receiver operating characteristic (ROC) curve (AUC), whereas calibration was assessed by the Hosmer–Lemeshow goodness-of-fit test and calibration plot generated through 1,000 bootstrap resamples for internal validation. All tests were two-tailed, and a *p* value < 0.05 was considered statistically significant.

## Results

### Maternal and neonatal characteristics by delayed lactogenesis II status

Among the 325 advanced-age women included in this study, 117 (36.0%) experienced delayed lactogenesis II (DLII), defined as onset of copious milk secretion occurring later than 72 h postpartum. As shown in Table 1, maternal age and pre-pregnancy body mass index (BMI) were significantly higher in the DLII group compared with those without delay (38.4 ± 2.4 vs. 37.6 ± 2.2 years, *p* = 0.008; 25.2 ± 3.5 vs. 24.2 ± 2.8 kg/m^2^, *p* = 0.011). A greater proportion of women with delayed lactation were primiparous (65.0% vs. 48.6%, *p* = 0.006) and had a slightly shorter gestational age at delivery (38.1 ± 1.1 vs. 38.6 ± 0.9 weeks, *p* = 0.001). Pregnancy-induced hypertension was more common among women with DLII (16.2% vs. 8.7%, *p* = 0.041), whereas the prevalence of gestational diabetes mellitus did not differ significantly between groups (*p* = 0.219). Regarding intrapartum variables, the DLII group had a lower rate of elective cesarean section (64.1% vs. 80.3%, *p* = 0.002), a longer mean operation time (58.1 ± 13.8 vs. 52.8 ± 11.6 min, *p* = 0.001), and greater estimated blood loss (452 ± 145 vs. 389 ± 126 mL, *p* = 0.003). Post-operative analgesia use was comparable between groups. Neonatal outcomes were generally favorable, but infants of mothers with delayed lactogenesis had slightly lower median 1-min Apgar scores (9 (8–10) vs. 9 (9–10), *p* = 0.012). Moreover, mothers with DLII were significantly less likely to initiate rooming-in within 24 h (72.6% vs. 88.0%, *p* = 0.001), to perform first breastfeeding within 24 h (41.9% vs. 78.8%, *p* < 0.001), or to engage in early skin-to-skin contact (43.6% vs. 66.3%, *p* < 0.001). Collectively, these findings suggest that delayed lactogenesis among advanced-age cesarean mothers is associated with higher maternal age and BMI, primiparity, hypertensive disorders, longer and more complicated surgeries, and suboptimal early breastfeeding behaviors. Information on exclusive breastfeeding rate at discharge was not consistently recorded in extractable fields in the electronic medical record system during the study period and therefore could not be reliably analyzed.

**Table 1:** Baseline characteristics of 325 advanced-age women undergoing cesarean section according to delayed lactogenesis II status.

Variables	Overall (*n* = 325)	No delay (*n* = 208)	Delay > 72 h (*n* = 117)	*p* value
Maternal characteristics				
Age, years	37.9 ± 2.3	37.6 ± 2.2	38.4 ± 2.4	0.008 **
Pre-pregnancy BMI, kg/m^2^	24.6 ± 3.1	24.2 ± 2.8	25.2 ± 3.5	0.011 *
Primiparous, *n* (%)	177 (54.5)	101 (48.6)	76 (65.0)	0.006 **
Gestational weeks	38.4 ± 1.0	38.6 ± 0.9	38.1 ± 1.1	0.001 **
Pregnancy-induced hypertension, *n* (%)	37 (11.4)	18 (8.7)	19 (16.2)	0.041 *
Gestational diabetes mellitus, *n* (%)	53 (16.3)	30 (14.4)	23 (19.7)	0.219
Intrapartum and surgical data				
Elective cesarean, *n* (%)	242 (74.5)	167 (80.3)	75 (64.1)	0.002 **
Regional anesthesia (vs general), *n* (%)	296 (91.1)	194 (93.3)	102 (87.2)	0.083
Operation time, min	54.7 ± 12.8	52.8 ± 11.6	58.1 ± 13.8	0.001 **
Estimated blood loss, mL	412 ± 135	389 ± 126	452 ± 145	0.003 **
Post-operative analgesia pump use, *n* (%)	287 (88.3)	188 (90.4)	99 (84.6)	0.126
Neonatal and feeding variables				
Birth weight, g	3,308 ± 402	3,324 ± 396	3,280 ± 412	0.356
1-min Apgar score	9 (9–10)	9 (9–10)	9 (8–10)	0.012 *
Rooming-in within 24 h, *n* (%)	268 (82.5)	183 (88.0)	85 (72.6)	0.001 **
First breastfeeding within 24 h, *n* (%)	213 (65.5)	164 (78.8)	49 (41.9)	<0.001 ***
Early skin-to-skin contact, *n* (%)	189 (58.2)	138 (66.3)	51 (43.6)	<0.001 ***

Data are presented as mean ± standard deviation (SD), median (interquartile range, IQR), or number (percentage) as appropriate. Comparisons between groups were performed using the independent samples *t* test or Mann–Whitney *U* test for continuous variables and the *χ*^2^ test for categorical variables. *p* values < 0.05 were considered statistically significant. BMI, body mass index; DLII, delayed lactogenesis II. Elective cesarean section was scheduled before labor onset/rupture of membranes; emergency cesarean section was performed intrapartum for urgent indications. *p < 0.05; **p < 0.01; ***p < 0.001.

### Logistic regression analysis of factors associated with delayed lactogenesis II

In the univariate analysis, several maternal and perinatal factors were significantly associated with delayed lactogenesis II (DLII) among advanced-age women undergoing cesarean section (Table 2). Multicollinearity diagnostics indicated no evidence of problematic collinearity among candidate predictors (all VIFs < 5). Older maternal age, higher pre-pregnancy body mass index (BMI), primiparity, shorter gestational age, pregnancy-induced hypertension, longer operation time, greater estimated blood loss, and lower 1-min Apgar scores were all linked to an increased likelihood of DLII (all *p* < 0.05). In contrast, elective cesarean delivery and early postpartum practices, including rooming-in within 24 h, breastfeeding initiation within 24 h, and early skin-to-skin contact, were associated with lower odds of delayed lactogenesis II. However, pregnancy-induced hypertension did not remain statistically significant in the multivariable model (aOR = 1.85, 95% CI 0.86–3.96; *p* = 0.113). Multivariate logistic regression identified maternal age (adjusted OR = 1.15, 95% CI 1.01–1.32, *p* = 0.036), pre-pregnancy BMI (aOR = 1.10, 95% CI 1.00–1.22, *p* = 0.049), primiparity (aOR = 1.74, 95% CI 1.02–2.96, *p* = 0.041), and shorter gestational age (aOR = 0.71, 95% CI 0.55–0.91, *p* = 0.007) as independent risk factors for DLII. Furthermore, elective cesarean section (aOR = 0.55, 95% CI 0.31–0.96, *p* = 0.037), rooming-in within 24 h (aOR = 0.49, 95% CI 0.27–0.89, *p* = 0.018), first breastfeeding within 24 h (aOR = 0.32, 95% CI 0.18–0.56, *p* < 0.001), and early skin-to-skin contact (aOR = 0.53, 95% CI 0.30–0.92, *p* = 0.026) remained significant protective factors. These findings suggest that both maternal characteristics and early postpartum behaviors contribute substantially to the risk of delayed lactogenesis among older cesarean mothers.

**Table 2:** Univariate and multivariate logistic regression analyses of factors associated with delayed lactogenesis II among 325 advanced-age women undergoing cesarean section.

Variables	Univariate OR (95% CI)	*p* value	Multivariate aOR (95% CI)	*p* value
Maternal age (per year)	1.18 (1.04–1.33)	0.008 **	1.15 (1.01–1.32)	0.036 *
Pre-pregnancy BMI (per kg/m^2^)	1.12 (1.02–1.24)	0.015 *	1.10 (1.00–1.22)	0.049 *
Primiparity (vs multiparity)	1.98 (1.24–3.15)	0.004 **	1.74 (1.02–2.96)	0.041 *
Gestational age (per week)	0.68 (0.53–0.87)	0.002 **	0.71 (0.55–0.91)	0.007 **
Pregnancy-induced hypertension (vs none)	2.03 (1.01–4.10)	0.046 *	1.85 (0.86–3.96)	0.113
Elective cesarean (vs emergency)	0.46 (0.28–0.75)	0.002 **	0.55 (0.31–0.96)	0.037 *
Operation time (per 10 min)	1.26 (1.10–1.45)	0.001 **	1.21 (1.05–1.41)	0.010 *
Estimated blood loss (per 100 mL)	1.22 (1.07–1.38)	0.003 **	1.18 (1.03–1.35)	0.016 *
1-min Apgar (per score)	0.79 (0.64–0.97)	0.024 *	0.84 (0.66–1.06)	0.145
Rooming-in within 24 h (yes vs no)	0.41 (0.24–0.70)	0.001 **	0.49 (0.27–0.89)	0.018 *
First breastfeeding within 24 h (yes vs no)	0.25 (0.15–0.41)	<0.001 ***	0.32 (0.18–0.56)	<0.001 ***
Early skin-to-skin contact (yes vs no)	0.44 (0.27–0.71)	<0.001 ***	0.53 (0.30–0.92)	0.026 *

Data are presented as odds ratios (OR) with 95% confidence intervals (CI). Variables with *p* < 0.10 in univariate analysis were entered into the multivariate logistic regression model. Adjusted odds ratios (aOR) were calculated using a backward stepwise method to identify independent predictors of delayed lactogenesis II (DLII). *P* < 0.05 was considered statistically significant. BMI, body mass index; DLII, delayed lactogenesis II; OR, odds ratio; aOR, adjusted odds ratio; CI = confidence interval. *p < 0.05; **p < 0.01; ***p < 0.001.

### Model performance and validation

**Figure 1:**
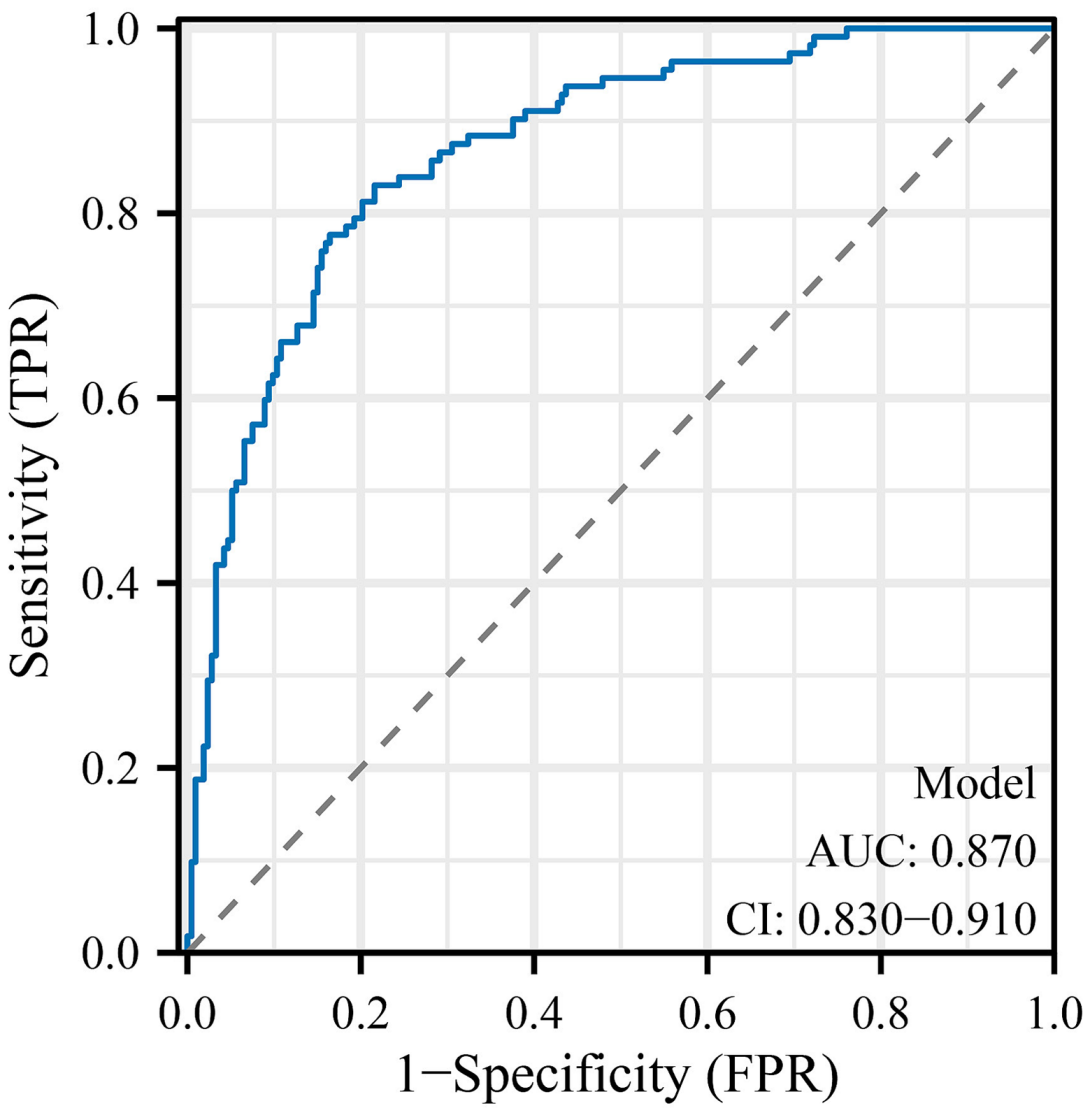
Receiver operating characteristic (ROC) curve for the prediction model of delayed lactogenesis II. The ROC curve illustrates the discriminative performance of the prediction model for delayed lactogenesis II (DLII) among advanced-age women undergoing cesarean section. The area under the curve (AUC) was 0.870 (95% CI 0.830–0.910), indicating excellent discrimination between participants with and without DLII. The diagonal dashed line represents the reference line of random prediction (AUC = 0.5).

**Figure 2:**
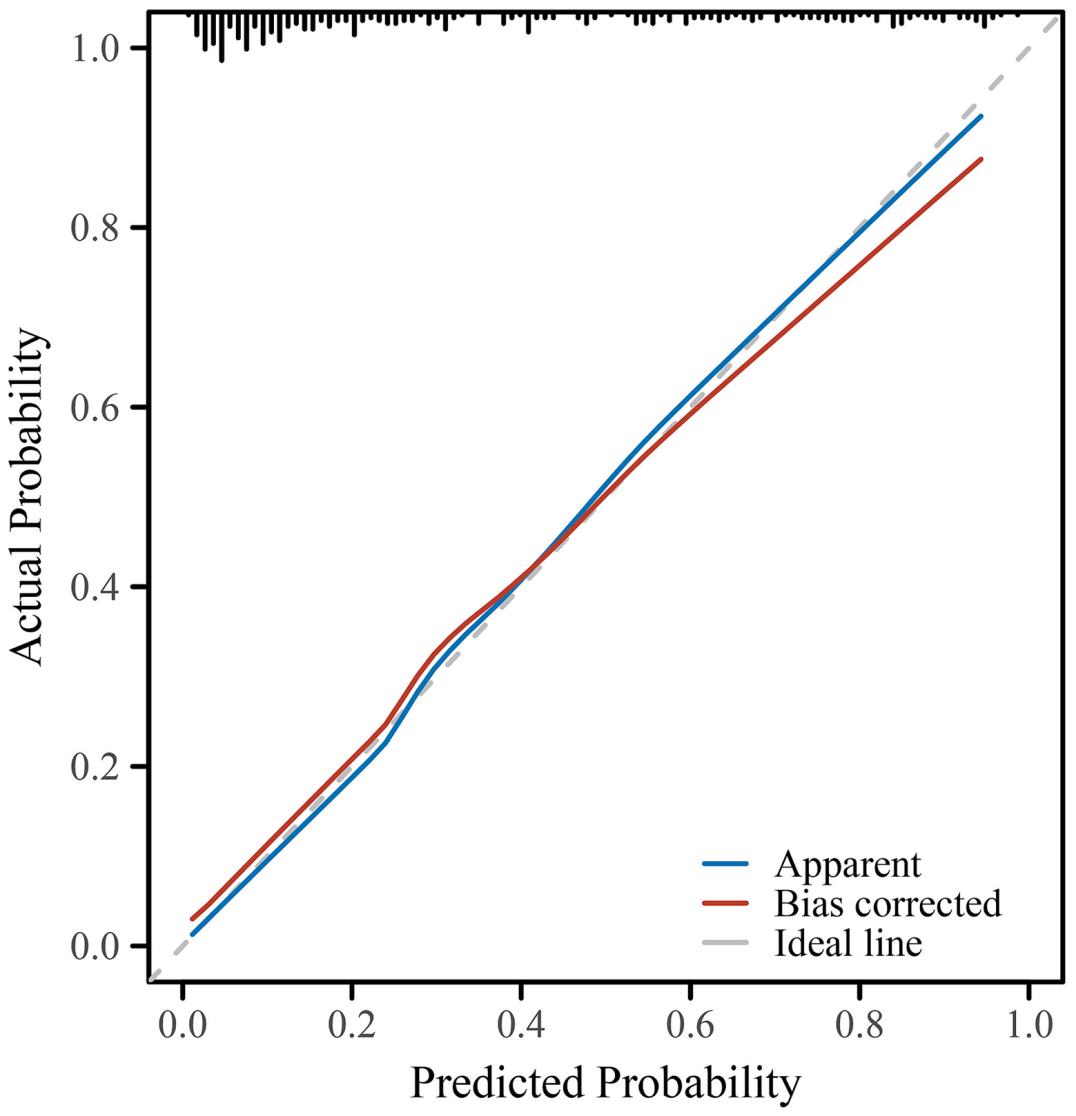
Calibration curve of the prediction model for delayed lactogenesis II. Calibration of the model was assessed using 1,000 bootstrap resamples. The blue line represents the apparent performance of the model, the red line denotes the bias-corrected estimates, and the gray dashed line indicates the ideal line of perfect agreement between predicted and observed probabilities. The close overlap between the bias-corrected and ideal lines demonstrates good model calibration (Hosmer–Lemeshow *χ*^2^ = 5.60, *p* = 0.692).

**Figure 3:**
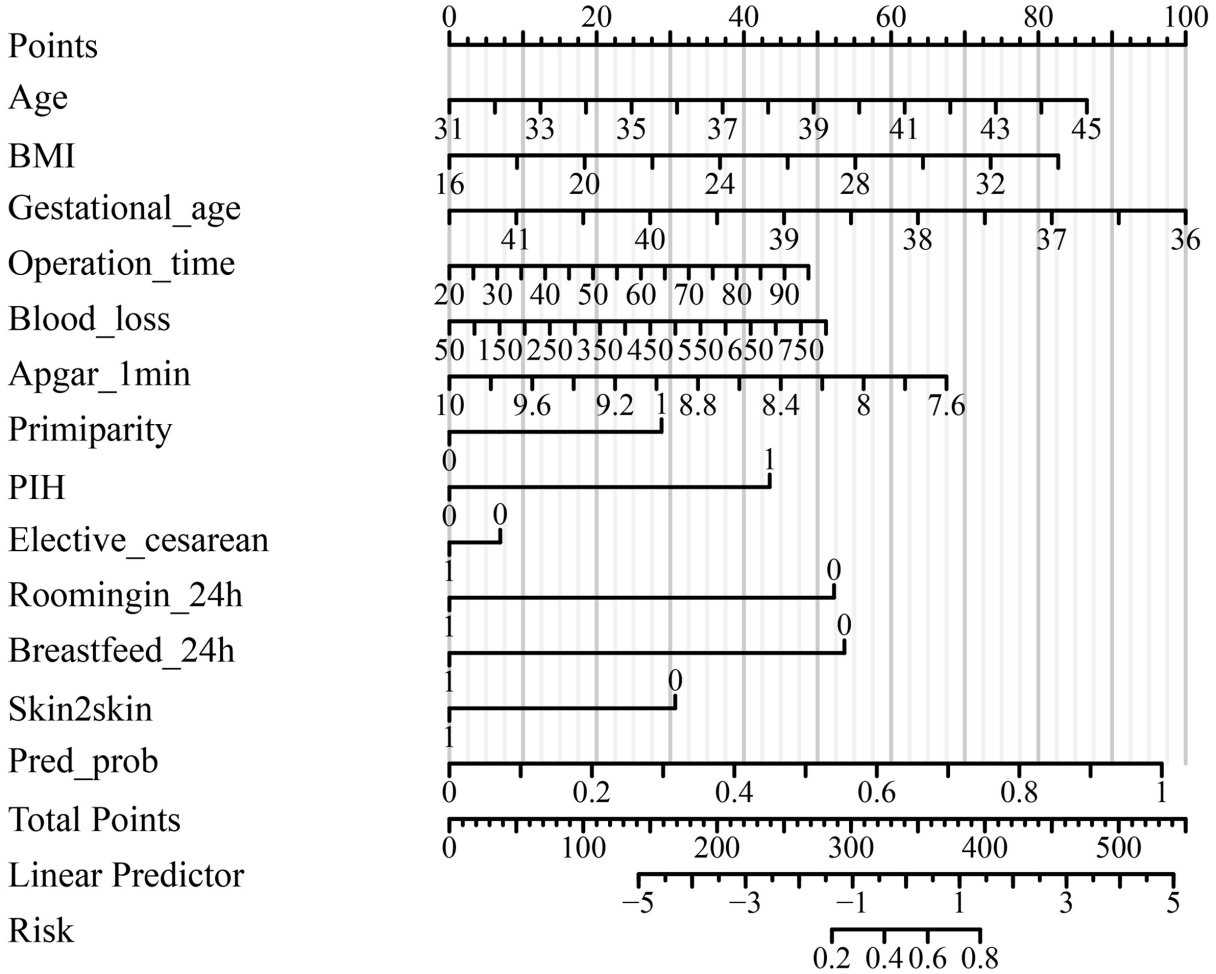
Nomogram for predicting delayed lactogenesis II among advanced-age women undergoing cesarean section. The nomogram integrates independent predictors identified in multivariate logistic regression, including maternal age, pre-pregnancy body mass index (BMI), gestational age, operation time, estimated blood loss, 1-min Apgar score, primiparity, pregnancy-induced hypertension (PIH), elective cesarean section, rooming-in within 24 h, first breastfeeding within 24 h, and early skin-to-skin contact. Each variable corresponds to a specific point value, and the total score (sum of all points) is projected onto the bottom scale to estimate the individual probability of delayed lactogenesis II.

The predictive model for delayed lactogenesis II (DLII) demonstrated excellent discriminative ability and good calibration (Table 3; Figures 1–3). As shown in the receiver operating characteristic (ROC) curve (Figure 1), the area under the curve (AUC) was 0.870 (95% CI 0.830–0.910), indicating strong discrimination between women with and without DLII. The likelihood ratio test confirmed that the overall model was statistically significant (*χ*^2^ = 142.11, *p* < 0.001), suggesting that the included predictors contributed meaningfully to model performance. Calibration analysis further supported the model’s reliability: the Hosmer–Lemeshow goodness-of-fit test yielded *χ*^2^ = 5.60 with *p* = 0.692, indicating good agreement between predicted and observed probabilities. In the calibration plot (Figure 2), the bias-corrected line closely overlapped with the ideal diagonal line, showing minimal deviation and excellent model fit. The constructed nomogram (Figure 3) visually represents the contribution of each independent predictor—including maternal age, pre-pregnancy BMI, gestational age, operative characteristics, and early postpartum behaviors—to the overall risk score, facilitating individualized clinical prediction and bedside applicability. Together, these findings confirm that the developed model possesses high predictive accuracy, strong internal validity, and good clinical interpretability for identifying advanced-age cesarean mothers at risk of delayed lactogenesis II.

**Table 3:** Model performance and validation results of the prediction model for delayed lactogenesis II.

Evaluation index	Statistical indicator	Value (95% CI)	*p* value/Interpretation
Discrimination	AUC (C-index)	0.870 (0.830–0.910)	Excellent discriminative ability
Model fit	Likelihood ratio *χ*^2^	142.11	< 0.001
Calibration	Hosmer–Lemeshow *χ*^2^	5.60	0.692 (good fit)

AUC, area under the receiver operating characteristic curve; CI, confidence interval. Model discrimination was evaluated using AUC and C-index; calibration was assessed using the Hosmer–Lemeshow goodness-of-fit test. AUC values between 0.7 and 0.9 indicate good discriminative ability, while *p* > 0.05 in the Hosmer–Lemeshow test denotes satisfactory model calibration.

## Discussion

The increasing prevalence of advanced maternal age, commonly defined as childbirth at 35 years or older (17), together with persistently high rates of cesarean delivery, has drawn increasing attention to lactation challenges in this population. Delayed lactogenesis II (DLII), defined as the delayed onset of copious milk secretion beyond 72 h postpartum (5), represents a well-recognized barrier to successful breastfeeding initiation. DLII has been associated with early breastfeeding difficulties and suboptimal infant feeding practices, which may contribute to neonatal dehydration, hypoglycemia, and early formula supplementation (18). In addition, delayed onset of lactation may increase maternal anxiety and undermine breastfeeding confidence, potentially affecting early mother–infant interaction and continuity of breastfeeding support (7). Despite the recognized importance of timely lactogenesis, risk factors and predictive indicators of DLII among older women undergoing cesarean section remain insufficiently characterized, underscoring the need for tailored risk stratification and targeted interventions.

In response to this clinical need, the present investigation retrospectively analyzed a cohort of 325 advanced maternal age women who underwent cesarean delivery, revealing a substantial DLII incidence of 36.0%, which is comparable to the prevalence of approximately 31% reported among women of advanced maternal age in recent studies (6). Employing multivariate logistic regression, the study identified key maternal and perinatal determinants—including elevated pre-pregnancy BMI, advanced maternal age, primiparity, and shorter gestational duration—as well as modifiable early postpartum behaviors such as the timing of mother-infant contact and initiation of breastfeeding. These findings informed the development of a robust nomogram with excellent discriminatory capacity (AUC = 0.870), facilitating individualized risk prediction for DLII. By integrating multifactorial influences, this work advances the precision of clinical assessment and lays the foundation for proactive lactation support strategies tailored to high-risk older cesarean-delivered mothers.

In our cohort, advanced maternal age, higher pre-pregnancy BMI, primiparity, and shorter gestational age were independently associated with DLII, whereas pregnancy-induced hypertension showed a univariable association but did not remain significant after multivariable adjustment, suggesting potential confounding or mediation by related maternal and peripartum factors. Regarding maternal characteristics, primiparity may contribute to delayed lactogenesis through several behavioral and psychosocial mechanisms. Previous studies have suggested that primiparity may be associated with challenges in breastfeeding technique, increased childbirth-related anxiety, and delayed establishment of effective milk removal, all of which could contribute to delayed lactogenesis II (6). Advanced maternal age and elevated BMI may impair timely lactogenesis through cumulative effects on mammary gland differentiation, metabolic regulation, and hormonal sensitivity, particularly involving prolactin and oxytocin signaling pathways (19, 20). Elevated BMI prior to pregnancy is known to disrupt normal mammary gland development and is associated with chronic low-grade inflammation and insulin resistance, both of which may negatively affect the hormonal milieu necessary for the timely onset of copious milk secretion (21). Although PIH was not an independent predictor in the adjusted model, it may still serve as a marker of a more complex perinatal profile. For instance, hypertensive disorders are associated with endothelial dysfunction and altered placental perfusion, which could theoretically influence lactogenesis-related hormonal and metabolic pathways (22). It is noteworthy that the finding of shorter gestational age in the DLII group resonates with reports indicating that even modest reductions in gestational length may delay the maturation of lactogenic hormonal cascades and mammary alveolar differentiation (5). Compared to previous studies that predominantly focused on Western populations, our findings in an Asian cohort reinforce the universality of these risk factors, while also highlighting subtle differences in their relative impact, possibly attributable to genetic and environmental modifiers (23, 24). Collectively, these observations substantiate a multifactorial physiological basis for DLII in older cesarean parturients, with each risk factor converging on the disruption of mammary gland priming and hormonal activation.

Building upon the distinct impact of peripartum management, our results reveal that non-elective surgery, prolonged operative time, and increased intraoperative blood loss are all associated with a higher incidence of DLII, underscoring the significance of surgical stress and perinatal hemodynamics in modulating the lactogenic process. Acute surgical stress is known to elicit a robust hypothalamic–pituitary–adrenal (HPA) axis response, elevating circulating cortisol which in turn can inhibit oxytocin release and impede milk ejection reflexes (25). Accordingly, the contrast observed between elective and emergency cesarean delivery in our model is best interpreted as reflecting these physiologic stress responses and care-related differences, rather than evidence of an inherent advantage associated with elective surgery. Prolonged surgery and greater blood loss may further exacerbate maternal stress responses and transient hypovolemia, potentially compromising mammary gland perfusion and delaying the postpartum hormonal switch essential for lactogenesis (26). Importantly, the markedly lower rates of early mother-infant skin-to-skin contact, rooming-in, and initial breastfeeding within 24 h in the DLII group provide a compelling clinical correlate; immediate postpartum mother-infant interaction is recognized to stimulate both prolactin and oxytocin release, thereby facilitating mammary glandular activation and milk synthesis (27). International randomized and multicenter studies have generally reported that early and frequent maternal–infant interaction accelerates lactogenesis, independent of delivery mode, although the effect size may be modulated by cultural practices and institutional protocols (28). Taken together, these findings suggest two complementary pathways: a physiologic stress pathway related to intrapartum and surgical stress responses, and a care-related pathway reflecting delays or disruptions in early breastfeeding-supportive practices. We emphasize that these pathways are conceptually distinct but may co-occur in clinical practice. Information on neonatal formula supplementation during the period of delayed breastfeeding initiation was not consistently available in structured records and therefore could not be incorporated into the present analyses, which should be addressed in future prospective studies. Clinically, this distinction is important because physiologic stressors may be less modifiable at the time of urgent delivery, whereas care-related factors are more directly targetable through standardized postpartum lactation support.

An important finding of this study was the observed difference in the likelihood of delayed lactogenesis II between elective and emergency cesarean delivery. From a physiologic perspective, emergency cesarean delivery commonly occurs in the setting of heightened intrapartum stress, which may activate the HPA axis and increase circulating cortisol, potentially blunting oxytocin release and disrupting the early prolactin–oxytocin dynamics required for timely lactogenesis II. In addition, hemodynamic instability, longer procedures, and greater blood loss may further delay the postpartum endocrine transition that supports copious milk secretion. It should be noted that early postpartum practices, including skin-to-skin contact, rooming-in, and breastfeeding initiation within 24 h, may occur downstream of cesarean urgency and thus may partially mediate the relationship between emergency cesarean delivery and delayed lactogenesis II. Accordingly, adjustment for these variables may result in overadjustment, and the estimated association for cesarean urgency in our multivariable model should be interpreted as a conditional (direct) association accounting for early postpartum care practices, rather than the total association of cesarean urgency with delayed lactogenesis II. Given the retrospective observational design, this finding should not be interpreted as a direct effect of surgical urgency itself. In addition, emergency cesarean delivery is frequently preceded by prolonged or complicated labor, rupture of membranes, labor induction or augmentation, maternal exhaustion, or intrapartum infection, none of which were systematically measured or available for adjustment in this retrospective analysis. These unmeasured intrapartum factors may represent important sources of residual confounding and should be considered when interpreting the observed association between cesarean urgency and delayed lactogenesis II. While cesarean delivery overall has been linked to delayed lactogenesis, prior evidence suggests heterogeneity by surgical urgency: unscheduled or emergency cesarean delivery has been associated with delayed onset of lactation and poorer early breastfeeding outcomes compared with planned procedures, which is more plausibly explained by differences in intrapartum stress exposure and early postpartum care practices that commonly accompany emergency cesarean delivery (29). In our cohort, emergency cesarean deliveries were also accompanied by longer operative time, greater blood loss, and lower early postpartum contact behaviors, all of which plausibly impede oxytocin/prolactin-mediated lactation initiation. Conversely, elective cesarean delivery is typically performed under more controlled conditions (e.g., stable maternal status and preoperative preparation), which may make it easier to implement breastfeeding-supportive practices (skin-to-skin contact, rooming-in, and early breastfeeding initiation) in a timely manner. We acknowledge that some studies have reported delayed breastfeeding behaviors even after planned cesarean delivery, underscoring that institutional protocols and postpartum care pathways may modify this relationship. Therefore, the observed association should be interpreted as reflecting a cluster of physiologic stressors and care disruptions surrounding emergency cesarean delivery, highlighting the need for earlier and more intensive lactation support in this group rather than implying any inherent benefit of elective surgery (7).

With respect to the role of anesthesia and postoperative analgesia, our data indicate no significant difference in DLII incidence based on the type of anesthesia or the perioperative analgesic regimen employed. This aligns with existing literature suggesting that, while general and regional anesthesia may differentially affect maternal consciousness and stress, neither appears to exert a consistent direct effect on the neuroendocrine pathways governing lactogenesis when standardized perioperative protocols are followed (30, 31). Experimental evidence suggests that anesthetic agents can transiently inhibit prolactin and oxytocin secretion, but these effects are generally short-lived and may be outweighed by the benefits of effective pain control, which itself reduces maternal stress responses (32). Similarly, the use of multimodal analgesia post-cesarean has not been shown to adversely affect early lactation outcomes (33). Notably, the lack of association between anesthesia-related factors and DLII in our multivariable model supports the contention that standardized analgesic management may mitigate the indirect impact of perioperative stress on lactogenesis (34). This finding is particularly relevant in light of ongoing debates regarding the optimal anesthesia strategies for cesarean delivery in older and high-risk populations.

The development and validation of a logistic regression-based risk prediction model for DLII, with high discriminatory performance and robust calibration, represents a methodological advancement in individualized risk stratification. Mechanistically, the inclusion of variables such as maternal age, BMI, parity, gestational age, and peripartum management factors reflects the integration of distinct yet interacting biological and clinical pathways implicated in lactogenesis disruption (5). Compared to prior models predicting cesarean or perinatal complications, which often emphasize demographic and obstetric history (31), our model uniquely incorporates modifiable perioperative interventions and early postpartum care variables, thus offering actionable targets for clinical decision-making. Internationally, most published DLII risk models have been derived from smaller, often homogeneous cohorts, and rarely validated in populations with high rates of advanced maternal age and cesarean delivery (32). The present approach, combining rigorous statistical methodology with clinically relevant predictors, advances existing frameworks by facilitating early identification and targeted intervention for high-risk individuals.

Finally, advanced maternal age, increased pre-pregnancy BMI, primiparity, and shorter gestational duration were independently associated with delayed lactogenesis II, whereas differences in cesarean urgency and early postpartum care practices were associated with variation in the likelihood of delayed lactogenesis II. Consistent with this interpretation, early maternal–infant interactions have been mechanistically linked to rapid activation of lactogenic hormone cascades and mammary glandular priming (34). Comparative analyses of risk factor profiles across populations and delivery modes reveal both universal patterns and context-specific modifiers, such as the influence of psychosocial support and institutional breastfeeding policies (35). This nuanced understanding underscores the necessity of integrated, individualized strategies to optimize lactogenesis in high-risk cesarean populations.

Despite the robust design and rigorous internal validation of our predictive model, several limitations merit consideration. First, the retrospective nature and reliance on a single-center clinical database may introduce information bias and limit the granularity of certain variables, such as the qualitative aspects of breastfeeding support and maternal psychological status, which are not routinely documented and may substantially affect lactogenesis. Second, several intrapartum factors that commonly precede emergency cesarean delivery—such as duration of labor, rupture of membranes, induction or augmentation of labor, maternal exhaustion, and intrapartum infection—were not available in structured form and could not be adjusted for, which may have introduced residual confounding. Third, the exclusion of women with severe obstetric complications, multiple pregnancies, or incomplete data—while necessary for analytic clarity—may result in a study population that underrepresents higher-risk groups and thus restricts the external generalizability of our findings to broader obstetric populations, particularly in settings with more diverse clinical profiles. Fourth, neonatal characteristics were not comprehensively captured in our extractable dataset. Specifically, some neonatal parameters (e.g., the 5-min Apgar score and other routinely reported neonatal clinical indicators) were not consistently recorded in structured fields and therefore could not be analyzed, which may have limited neonatal risk characterization. In addition, breastfeeding outcomes beyond DLII (e.g., exclusive breastfeeding at discharge and post-discharge breastfeeding continuation) were not consistently available in structured fields, precluding a more complete description of feeding patterns.

In summary, this study identified advanced maternal age, elevated pre-pregnancy BMI, primiparity, and shorter gestational age as independent risk factors for delayed lactogenesis II among advanced-age cesarean mothers, while cesarean urgency and early postpartum care practices were associated with variation in the likelihood of delayed lactogenesis II. In addition, emergency cesarean delivery should be viewed as a marker of a higher-risk intrapartum and early postpartum context that warrants enhanced lactation support. The developed nomogram demonstrates high discriminative power and clinical applicability, offering a valuable tool for personalized risk assessment and targeted intervention. Future research should prioritize prospective, multicenter validation and integrate psychosocial and healthcare system factors to enhance model generalizability and inform the development of comprehensive post-cesarean lactation support strategies.

## Data Availability

The original contributions presented in the study are included in the article/supplementary material, further inquiries can be directed to the corresponding author.
